# Supplementary material for: Nanoparticles for Thrombolytic Therapy in Ischemic Stroke: A Systematic Review and Meta-Analysis of Preclinical Studies
Source: Pharmaceutics. 2025 Feb 6;17(2):208. doi: 10.3390/pharmaceutics17020208 (PMC11859612; doi:10.3390/pharmaceutics17020208)
Supplement: Supplementary file 1 [file pharmaceutics-17-00208-s001.zip › pharmaceutics-3431099-supplementary.pdf]

Supplementary Information

Table S1. PRISMA 2020 Checklist.

Section and Topic	Item #	Checklist item	Location where item is reported
**TITLE**			
Title	1	Identify the report as a systematic review.	Line 2-3
**ABSTRACT**			
Abstract	2	See the PRISMA 2020 for Abstracts checklist.	Line 24-45
**INTRODUCTION**			
Rationale	3	Describe the rationale for the review in the context of existing knowledge.	Line 110-119
Objectives	4	Provide an explicit statement of the objective(s) or question(s) the review addresses.	Line 110-119
**METHODS**			
Eligibility criteria	5	Specify the inclusion and exclusion criteria for the review and how studies were grouped for the syntheses.	Line 140-146
Information sources	6	Specify all databases, registers, websites, organisations, reference lists and other sources searched or consulted to identify studies. Specify the date when each source was last searched or consulted.	Line 125-131
Search strategy	7	Present the full search strategies for all databases, registers and websites, including any filters and limits used.	Line 125-135
Selection process	8	Specify the methods used to decide whether a study met the inclusion criteria of the review, including how many reviewers screened each record and each report retrieved, whether they worked independently, and if applicable, details of automation tools used in the process.	Line 125-147
Data collection process	9	Specify the methods used to collect data from reports, including how many reviewers collected data from each report, whether they worked independently, any processes for obtaining or confirming data from study investigators, and if applicable, details of automation tools used in the process.	Line 137-158
Data items	10a	List and define all outcomes for which data were sought. Specify whether all results that were compatible with each outcome domain in each study were sought (e.g. for all measures, time points, analyses), and if not, the methods used to decide which results to collect.	Line 139-185
	10b	List and define all other variables for which data were sought (e.g. participant and intervention characteristics, funding sources). Describe any assumptions made about any missing or unclear information.	Line 139-185
Study risk of bias assessment	11	Specify the methods used to assess risk of bias in the included studies, including details of the tool(s) used, how many reviewers assessed each study and whether they worked independently, and if applicable, details of automation tools used in the process.	Line 177-185
Effect measures	12	Specify for each outcome the effect measure(s) (e.g. risk ratio, mean difference) used in the synthesis or presentation of results.	Line 198-202
Synthesis methods	13a	Describe the processes used to decide which studies were eligible for each synthesis (e.g. tabulating the study intervention characteristics and comparing against the planned groups for each synthesis (item #5)).	Line 139-147
	13b	Describe any methods required to prepare the data for presentation or synthesis, such as handling of missing summary statistics, or data conversions.	Line 198-220
	13c	Describe any methods used to tabulate or visually display results of individual studies and syntheses.	Line 216-220

Section and Topic	Item #	Checklist item	Location where item is reported
	13d	Describe any methods used to synthesize results and provide a rationale for the choice(s). If meta-analysis was performed, describe the model(s), method(s) to identify the presence and extent of statistical heterogeneity, and software package(s) used.	Line 198-220
	13e	Describe any methods used to explore possible causes of heterogeneity among study results (e.g. subgroup analysis, meta-regression).	Line 210-213
	13f	Describe any sensitivity analyses conducted to assess robustness of the synthesized results.	Line 198-220
Reporting bias assessment	14	Describe any methods used to assess risk of bias due to missing results in a synthesis (arising from reporting biases).	Line 216-220
Certainty assessment	15	Describe any methods used to assess certainty (or confidence) in the body of evidence for an outcome.	Line 198-220
**RESULTS**			
Study selection	16a	Describe the results of the search and selection process, from the number of records identified in the search to the number of studies included in the review, ideally using a flow diagram.	Line 224-232
	16b	Cite studies that might appear to meet the inclusion criteria, but which were excluded, and explain why they were excluded.	Line 224-232, 254-257
Study characteristics	17	Cite each included study and present its characteristics.	Figure 3, Table S2
Risk of bias in studies	18	Present assessments of risk of bias for each included study.	Table S4
Results of individual studies	19	For all outcomes, present, for each study: (a) summary statistics for each group (where appropriate) and (b) an effect estimate and its precision (e.g. confidence/credible interval), ideally using structured tables or plots.	Figure 3
Results of syntheses	20a	For each synthesis, briefly summarise the characteristics and risk of bias among contributing studies.	Table S2, S4
	20b	Present results of all statistical syntheses conducted. If meta-analysis was done, present for each the summary estimate and its precision (e.g. confidence/credible interval) and measures of statistical heterogeneity. If comparing groups, describe the direction of the effect.	Figure 3, 4
	20c	Present results of all investigations of possible causes of heterogeneity among study results.	Line 502-511
	20d	Present results of all sensitivity analyses conducted to assess the robustness of the synthesized results.	Line 188-198
Reporting biases	21	Present assessments of risk of bias due to missing results (arising from reporting biases) for each synthesis assessed.	Line 331-335, Figure 7
Certainty of evidence	22	Present assessments of certainty (or confidence) in the body of evidence for each outcome assessed.	Line 200-222
**DISCUSSION**			
Discussion	23a	Provide a general interpretation of the results in the context of other evidence.	Line 341-350
	23b	Discuss any limitations of the evidence included in the review.	Line 481-497
	23c	Discuss any limitations of the review processes used.	Line 481-497
	23d	Discuss implications of the results for practice, policy, and future research.	Line 462-479

Section and Topic	Item #	Checklist item	Location where item is reported
**OTHER INFORMATION**			
Registration and protocol	24a	Provide registration information for the review, including register name and registration number, or state that the review was not registered.	N/A
	24b	Indicate where the review protocol can be accessed, or state that a protocol was not prepared.	N/A
	24c	Describe and explain any amendments to information provided at registration or in the protocol.	N/A
Support	25	Describe sources of financial or non-financial support for the review, and the role of the funders or sponsors in the review.	Line 533-537
Competing interests	26	Declare any competing interests of review authors.	Line 541
Availability of data, code and other materials	27	Report which of the following are publicly available and where they can be found: template data collection forms; data extracted from included studies; data used for all analyses; analytic code; any other materials used in the review.	Line 540

Figure S1. PRISMA 2020 Flow Diagram.

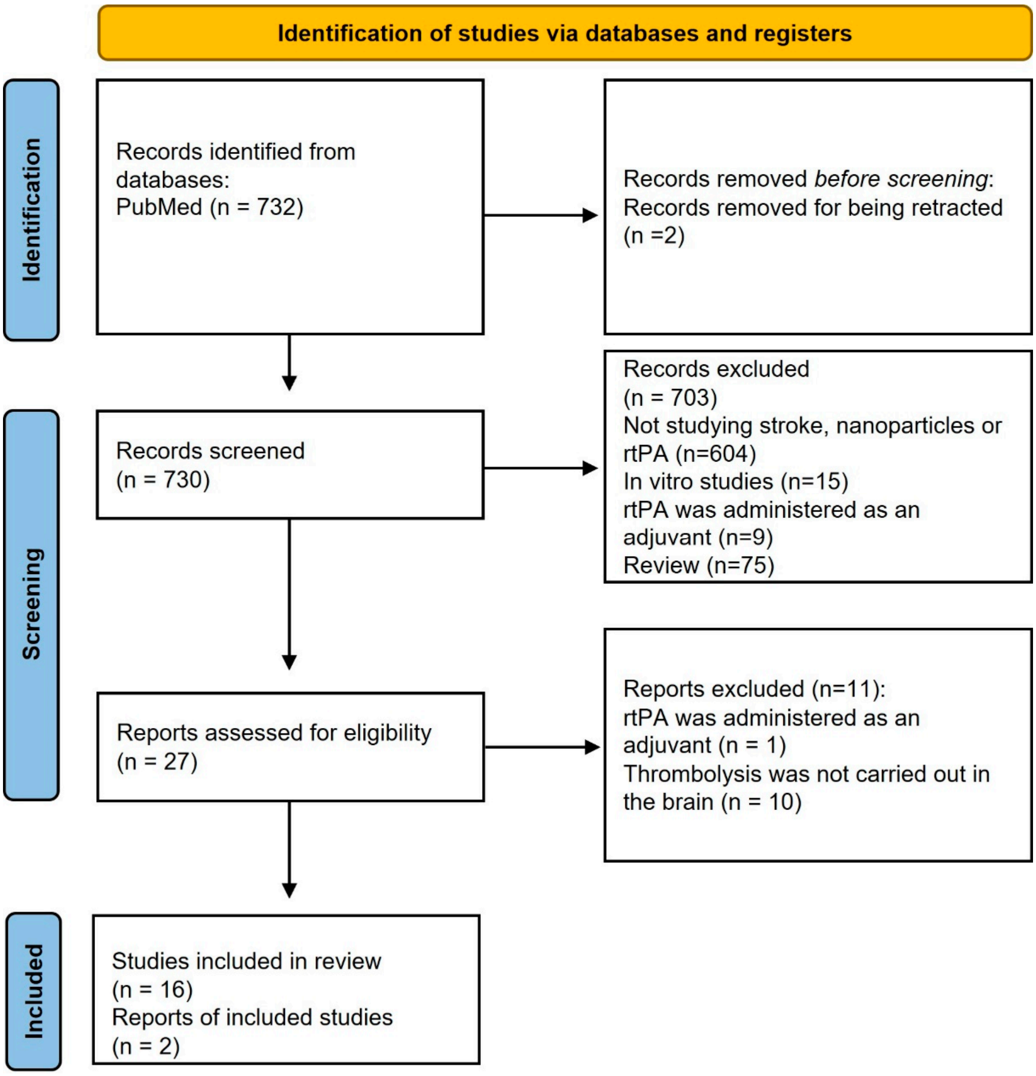

Table S2. PRIMED[2] items and their ratings.

**A. Sex of animals**	
0	Benefit shown in either male or female animals
2	Benefit shown in both male and female animals
**B. Age of animals**	
0	Benefit shown in only young animals
2	Benefit shown in older adult animals
**C. Species and strains of animals**	
0	Benefit shown in one rodent species and one strain of the species
1	Benefit shown in ≥2 rodent species or ≥2 strains of a species
2	Benefit shown in rodent plus primates
**D. Reproducibility**	
0	Benefit shown in one species in one laboratory
1	Benefit shown in ≥2 species but in one laboratory or in one species but ≥2 laboratories
2	Benefit shown in ≥2 species ≥2 laboratories
**E. Treatment time epoch**	
0	No significant benefit is shown
1	Benefit shown in one treatment time epoch
2	Benefit shown in ≥2 treatment time epochs
**F. Baseline comorbidities**	
0	Benefit shown in healthy animals only

1	Benefit shown in animals with one comorbid condition
2	Benefit shown in animals ≥2 comorbid conditions
**G. Feasible time window**	
0	Benefit shown only with treatment admninistration before ischemic onset, al ischemia onset, or within 45 minutes after onset of ischemia
2	Benefit shown with treatment administration 45 minutes or more following onset of ischemia
**H. Dose-response**	
0	Benefit is shown with only single dose
2	Benefit is shown with multiple doses with dose-response relationship
**I. Feasible route of delivery**	
0	Benefit is shown with delivery of the stimulation/drug via infeasible routes such as implanted deep electrodes or intraventricular injection of drug
1	Benefit is shown with delivery of the stimulation/drug via an external but somewhat infeasible route such as epicranial electrodes and intrarterial injection
2	Benefit is shown with delivery of the stimulation/drug via transcranial and intravenous routes
**J. Behavioral and/or long-term outcome assessment**	
0	Absence of benefit for neither behavioral nor long-term outcome
1	Benefit shown for either behavioral or long-term outcome (≥30 days after ischemia)
2	Benefit shown for both behavioral and long-term outcomes (≥30 days after ischemia)
**K. Typical infarct volume reduction magnitude**	
0	Small effect size (Cohen's d 0.2-0.39)
1	Medium effect size (Cohen's d 0.4-0.69)

2	Large effect size (Cohen's d≥ 0.7)
**Readiness-for-translation score (0-22)**	
0-7	Low readiness
8-15	Intermediate readiness
16-22	High readiness

Table S3. Summary of the studies divided into nanoparticles and cell membrane-derived nanomedicines.

First Author	Year	Specie	Animals	Stroke Model	Biomaterial and size	Combined with	rtPA dose	Treatment Timepoint(s)	Route	Lesion Volume	Functional Assessments
**Nanoparticles**											
Deng J et al.	2018	Rat	15 (n=5/group)	MCAo (filament)	PEG-PCL + rtPA (~130nm)	N/A	1 mg/kg	2 hours	IV	Yes (TTC at 24 hours)	Neurological score (18 points)
Hu J et al.	2018	Mouse	20 (n=4/group)	Thromboembolic model ($FeCl_3$ in MCA)	Magnetic microrods + rtPA (L=1.3μm, D=0.5 μm)	Magnets	1 mg/kg	30 minutes	IA	Yes (TTC at 24 hours)	N/A
Huang L et al.	2019	Mouse	24 (n=6/group)	Thromboembolic model ($FeCl_3$ in MCA)	PAA + MNP + rtPA	N/A	10 mg/kg	10 minutes	IV	Yes (TTC at 24 hours)	N/A
Mei T et al.	2019	Mouse	25 (n=5/group)	Photothrombotic Model (Rose Bengal)	Polyion complex + rtPA (~50nm)	N/A	1 mg/kg	10 minutes	IV	Yes (TTC at 24 hours)	Cylinder
Zenych A et al.	2021	Mouse	n≥5/group	Thromboembolic model (thrombin in MCA)	Polysacharide + fucoidan + rtPA (~700nm)	N/A	10 mg/kg	20 minutes	IV	Yes (MRI 24 hours)	N/A
Choi W et al.	2022	Mouse	33 (n=3-5/group)	Photothrombotic Model (Rose Bengal + thrombin)	Gold nanostars and nanospheres (~100nm)	US and microbubble	10 mg/kg	20 minutes	IV	Yes (TTC 24 hours)	N/A
Choi W et al.	2022	Mouse	30 (n=3-4/group)	Photothrombotic Model (Rose Bengal + thrombin)	PLGA + SPIONs + rtPA (3μm)	US	2 mg/kg	20 minutes, 3 hours	IV	Yes (TTC 24 hours)	Rotarod
Correa-Paz C et al.	2022	Mouse	45 (n=5/group)	Thromboembolic model (thrombin in MCA)	PDACMAC + PSS + Gelatin + iONs + rtPA (~800nm)	US	1 mg/kg	30 minutes	IV	Yes (MRI 24 hours)	N/A
Fournier L et al.	2023	Mouse	75 (n=18-20/group)	Thromboembolic model (thrombin in MCA)	Fucoidan + MBs + rtPA (~3μm)	US	1 mg/kg	30 minutes	IV	Yes (MRI 24 hours)	N/A

Yin J et al.	2024	Rat	30 (n=3/group)	MCAo (filament)	DNA + thrombin aptamer + rtPA (~70nm)	N/A	0.15 mg/kg	10 minutes, 1h, 3h and 6h	IV	Yes (TTC 24 hours)	Neurological score (5 points)
**Cell membrane-derived nanomedicines**											
Xu J et al.	2019	Mouse	30 (n=10/group)	MCAo (filament)	Platelet membrane + PLGA + rtPA (~170nm)	N/A	1 mg/kg	2 hours	IV	N/A	Neurological scores (5 points)
Xu J et al.	2019	Mouse	25 (n=5/group)	MCAo (filament)	Platelet membrane + ZL006e + rtPA (~170nm)	N/A	0.5 mg/kg	2 hours	IV	Yes (MRI and TTC at 24 hours)	Neurological scores (5 points)
Quan X et al.	2022	Mouse	15 (n=3/group)	Photothrombotic Model (Rose Bengal)	Platelet membrane + liposome + annexin V + rtPA (~140nm)	N/A	3 mg/kg	90 minutes	IV	Yes (MRI and LSI at 48 hours: TTC 7 at days)	Neurological score (14 points), Catwalk CT
Yu W et al.	2022	Mouse	21 (n=3/group)	Photothrombotic Model (Rose Bengal)	Melanin + platelet membrane + rtPA (~180nm)	N/A	1 mg/kg	20 minutes	IV	Yes (TTC 24 hours)	N/A
Quan X et al.	2023	Mouse	24 (n=3/group)	Photothrombotic Model (Rose Bengal)	Platelet membrane + adamantane + CB + ASA + rtPA (~160nm)	N/A	3 mg/kg	90 minutes	IV	Yes (MRI, LSI, OCT at 48 hours: TTC 7 at days)	Neurological score (8 points), Catwalk CT
Kong J et al.	2024	Rat	80 (n=10/group)	MCAo (filament)	Platelet membrane + zein + HO-Se-Se-OH, DHA (~220nm)	N/A	N/A	2 hours	IV	Yes (MRI at 7 days)	Morris water maze, beam walking, neurological score (14 points)
Migliavacca M et al.	2024	Mouse	56 (n=8/group)	Thromboembolic model (thrombin in MCA)	Platelet membrane (~200nm)	N/A	1 mg/kg	30 minutes	IV	Yes (MRI at 24 hours)	N/A

Quan X et al.	2024	Mouse	42 (n=3/group)	Photothrombotic Model (Rose Bengal)	Neutrophil + TUP + rtPA (~135nm)	NIR-II laser	10 mg/kg	90 minutes	IV	Yes (MRI at 72 hours)	Neurological score, Catwalk CT

Abbreviations: ASA, acetylsalicylic acid; CB, Cucurbit[7]uril; DHA, docosahexaenoic acid; iONs, iron oxide nanoparticles; IA, intraarterial; IV, intravenous; LSI, laser speckle imaging; MBs, microbubbles; MCA, middle cerebral artery; MCAo, middle cerebral artery occlusion; MNP, magnetic nanoparticles; MRI, magnetic resonance imaging; NIR-II, near-infrared II; OCT, optical coherence tomography; PAA, polyacrylic acid; PDACMAC, poly(diallyldimethylammonium chloride); PEG-PCL, poly(ethylene glycol)-poly(ε-caprolactone); PLGA, poly(lactic-co-glycolic) acid; PSS, poly (sodium 4-styrenesulfonate); rtPA, recombinant tissue plasminogen activator; SPIONs, superparamagnetic iron oxide nanoparticles; TTC, 2,3,5-triphenyltetrazolium chloride; TUP, thylakoid upconversion nanoparticle; US, ultrasound.

Table S4. PRIMED^2 tool applied to included studies in the meta-analysis.

Study	Year	A	B	C	D	E	F	G	H	I	J	K	Total
**Deng J et al.**	**2018**	0	0	0	0	1	0	2	0	2	1	2	**8**
**Hu J et al.**	**2018**	0	0	0	0	1	1	0	0	1	0	2	**5**
**Huang L et al.**	**2019**	0	0	0	0	1	0	0	0	2	0	1	**4**
**Mei T et al.**	**2019**	0	0	0	0	1	0	0	0	2	1	2	**6**
**Xu J et al.**	**2019**	0	0	0	0	1	0	2	0	2	1	0	**6**
**Xu J et al.**	**2019**	0	0	0	0	1	0	2	0	2	1	2	**8**
**Zenych A et al.**	**2021**	0	0	0	0	1	0	0	0	2	0	2	**5**
**Choi W et al.**	**2022**	0	0	0	0	1	0	0	0	2	0	2	**5**
**Choi W et al.**	**2022**	0	0	0	0	2	0	2	0	2	1	2	**9**
**Correa-Paz C et al.**	**2022**	0	0	0	0	0	0	0	0	2	0	2	**4**
**Quan X et al.**	**2022**	0	0	0	0	1	0	2	0	2	1	2	**8**
**Yu W et al.**	**2022**	0	0	0	0	1	0	0	0	2	0	2	**5**
**Fournier L et al.**	**2023**	0	0	0	0	1	0	0	0	2	0	2	**5**
**Quan X et al.**	**2023**	0	0	0	0	1	0	2	0	2	1	2	**8**
**Kong J et al.**	**2024**	0	0	0	0	1	0	2	0	2	1	2	**8**
**Migliavacca M et al.**	**2024**	0	0	0	0	0	0	0	0	2	0	1	**3**

**Quan X et al.**	**2024**	0	0	0	0	1	0	2	0	2	1	2	**9**
**Yin J et al.**	**2024**	0	0	0	0	2	0	2	0	2	1	2	**8**

PRIMED[2] items: (A) sex of animals; (B) age of animals; (C) species and strains of animals; (D) reproducibility; (E) treatment time epoch; (F) baseline comorbidities; (G) feasible time window; (H) dose-response; (I) feasible route of delivery; (J) behavioral and/or long-term outcome assessment; (K) typical infarct volume reduction magnitude.

Table S5. CAMARADES score of each included study.

Study	Year	A	B	C	D	E	F	G	H	I	J	Total
**Deng J et al.**	**2018**	1	1	0	0	0	1	0	0	1	1	**5**
**Hu J et al.**	**2018**	1	0	1	1	0	0	0	0	1	0	**4**
**Huang L et al.**	**2019**	1	1	0	0	0	1	0	0	1	1	**5**
**Mei T et al.**	**2019**	1	1	0	0	0	0	0	0	1	1	**4**
**Xu J et al.**	**2019**	1	0	1	0	0	0	0	0	1	1	**4**
**Xu J et al.**	**2019**	1	1	0	0	0	1	0	0	0	1	**4**
**Zenych A et al.**	**2021**	1	0	0	0	0	0	0	0	1	1	**3**
**Choi W et al.**	**2022**	1	0	0	0	0	1	0	0	1	1	**4**
**Choi W et al.**	**2022**	1	0	0	0	0	1	0	0	1	1	**4**
**Correa-Paz C et al.**	**2022**	1	1	0	0	0	1	0	0	1	1	**5**

**Quan X et al.**	**2022**	1	0	1	0	1	0	0	0	1	1	**5**
**Yu W et al.**	**2022**	1	0	0	0	0	1	0	0	1	1	**4**
**Fournier L et al.**	**2023**	1	0	0	0	0	0	0	0	1	1	**3**
**Quan X et al.**	**2023**	1	0	0	0	0	0	0	0	1	1	**3**
**Kong J et al.**	**2024**	1	0	1	0	0	0	0	0	1	1	**4**
**Migliavacca M et al.**	**2024**	1	1	0	0	0	1	0	0	1	1	**5**
**Quan X et al.**	**2024**	1	0	1	0	0	0	0	0	1	1	**4**
**Yin J et al.**	**2024**	1	1	1	1	1	0	0	0	1	1	**7**

CAMARADES items: (A) peer reviewed publication; (B) control of temperature; (C) random allocation to treatment or control; (D) blinded induction of ischemia; (E) blinded assessment of outcome; (F) use of anesthetic without significant intrinsic neuroprotective activity; (G) animal model (aged, diabetic, or hypertensive); (H) sample size calculation; (I) compliance with animal welfare regulations; and (J) statement of potential conflict of interests.
